# Identification of lysozyme in Venetin-1 nanoparticle from the coelomic fluid of the earthworm *Dendrobaena veneta*

**DOI:** 10.3389/fmolb.2025.1719519

**Published:** 2025-11-26

**Authors:** Marta J. Fiołka, Magdalena Dryglewska, Kinga Lewtak, Michał Rawski, Tomasz Buchwald, Ewa Skwarek, Sachin Kote, Jakub Faktor, Weronika Ścibek-Rejmontowska, Paulina Czaplewska, Wojciech Kaźmierski

**Affiliations:** 1 Department of Immunobiology, Institute of Biological Sciences, Faculty of Biology and Biotechnology, Maria Curie-Skłodowska University, Lublin, Poland; 2 Department of Rheumatology and Connective Tissue Diseases, Medical University of Lublin, Lublin, Poland; 3 SOLARIS National Synchrotron Radiation Centre, Jagiellonian University, Kraków, Poland; 4 Faculty of Materials Science and Technical Physics, Institute of Materials Research and Quantum Engineering, Poznań University of Technology, Poznań, Poland; 5 Department of Radiochemistry and Environmental Chemistry, Institute of Chemical Sciences, Maria Curie-Skłodowska University, Lublin, Poland; 6 International Center for Cancer Vaccine Science ICCVS, University of Gdańsk, Gdańsk, Poland; 7 Intercollegiate Faculty of Biotechnology, University of Gdańsk and Medical University of Gdańsk, Gdańsk, Poland; 8 Bruker Polska Sp. z o.o., Poznań, Poland

**Keywords:** Venetin-1, earthworms, coelomic fluid, lysozyme, lysenine, immunodetection

## Abstract

**Background:**

In the present study, lysozyme was analyzed in the nanoparticle Venetin-1 obtained from the coelomic fluid (CF) of the earthworm *Dendrobaena veneta*. Venetin-1 is a protein-polysaccharide complex and has proven anti-cancer (non-small lung cancer, colon cancer, cervical cancer), antifungal and immunostimulating properties.

**Methods:**

The studies were conducted using methods such as electrophoretic separation, immunodetection, and ELISA determination of lysozyme concentration. Spectroscopic methods such as FTIR and Raman spectroscopy were also employed. Proteomic analyses, SageELF separation, and zeta potential determination were also performed. Cryo-EM was used to determine the molecular structure of Venetin-1.

**Results:**

The lysozyme-type activity in this compound was significantly higher than in the crude CF. The analysis showed the highest concentration of lysozyme recognized by antibodies directed against human lysozyme is in the fraction containing compounds with a mass above 100 kDa and in the preparation obtained using a cut-off point of 6–8 kDa. Raman analysis showed a significant similarity of the protein part of Venetin-1 and EWL. FTIR analyses confirmed that the preparation obtained under these conditions showed high similarity to egg white lysozyme (EWL). Cryo-EM studies revealed the structure of the tested nanoparticle containing both lysozyme and lysenin fragments. Immunoblotting using antibodies directed against human lysozyme revealed proteins of mass 11, 33, 44, 88 and 132 kDa recognized in Venetin-1. Proteomic analyses confirmed the presence of these proteins and similarity to human lysozyme.

**Conclusion:**

These observations suggest the presence of polymeric forms of lysozyme in the tested complex, and zeta potential analysis revealed properties of the nanoparticle that predispose it to use in medicine.

## 1 Introduction

Before the discovery of penicillin in 1928, Alexander Fleming discovered lysozyme in 1922. Until his death in 1955, he was convinced that “the discovery of lysozyme will 1 day arouse more interest than the discovery of penicillin”. Although many may have a different opinion, in our opinion there is still much that is not known about the potential medical use of this interesting enzyme. The three-dimensional structure of lysozyme was analyzed by X-ray diffraction by David Phillips in 1965. He developed a model that was used to advance lysozyme research (Kirby, 2018).

Lysozyme (or muramidase, or N-acetylmuramic acid hydrolase E.C. 3.2.1.17) is a protein that exerts its enzymatic activity through the hydrolysis of the β-1,4-glycosidic bonds between N-acetylmuramic acid and N-acetylglucosamide in the peptidoglycan of the cell wall of Gram-positive bacteria (Ferraboschi et al., 2021). Additionally, lysozyme can inhibit or kill bacteria in a non-enzymatic manner. Its highly cationic nature promotes interactions with components of the bacterial cell wall, which lead to the activation of bacterial enzymes - autolysins, which in turn can degrade the cell wall (Hodges et al., 2012). Due to its broad range of antimicrobial activity, high physical and chemical stability, and proven safety, lysozyme from chicken egg white is used as a food preservative (Hodges et al., 2012). Lysozyme is the only biologically active ingredient that is on the list of permitted food additives. In many countries, lysozyme is used on an industrial scale as a preservative for meat products, fish, seafood, and dairy products (Khorshidian et al., 2022; Nawaz et al., 2022).

Lysozymes occur not only in human organs, tissues, and secretions, including tears, saliva, milk, and mucus (Nawaz et al., 2022), but also in organs, body fluids, and secretions of other vertebrates and invertebrates. The enzyme is also present in bacteria and plant tissues. Lysozymes are classified into three types: chicken type (type c), goose type (type g), and invertebrate type (type i) (Callewaert and Michiels, 2010); phage type, bacterial type, and plant type lysozyme can also be distinguished (Jollès and Jollès, 1984; Beintema and van Scheltinga, 1996; Fastrez, 1996).

Lysozymes also have antifungal (Ferraboschi et al., 2021) and antiviral effects, but the latter effect is related to their charge but not to their lytic activity (Losso et al., 2000). This enzyme has been shown to destroy the HIV virus (Singh et al., 2005). Lysozymes are also well-known immunomodulatory, immunostimulating, and antitumour agents (Mine and Kovacs-Nolan, 2004). This enzyme is a major component of many ethnic medicines listed in the Compendium of Materia Medica, a Ming Dynasty medical textbook. These preparations were made from the seeds of *Pithecellobium dulce*, soft-shell turtle, and sea cucumber (Araki et al., 1998; Xia and Wang, 2015; Yang and Bai, 2015; Gálvez-Iriqui et al., 2020). Type *i* lysozyme from sea cucumber, as a major immune factor of this organism, has been shown to have the ability to enhance human immunity (Cong et al., 2009), and lysozyme found in *P. dulce* seeds has been shown to be an effective antifungal agent (Gálvez-Iriqui et al., 2020).

Invertebrate animal models contribute to the development of complementary and alternative medicine. Earthworms are a very convenient research model. Such invertebrates as these annelids are inexpensive, ethically uncontroversial, and useful for elucidation of mechanisms underlying biological processes (Cooper et al., 2012). The use of pharmaceuticals derived from earthworms is highly developed in China and other Asian countries and is considered green medicine. Preparations prepared from these invertebrates are used to treat many diseases. Annelids are a known source of bioactive compounds, and their coelomic fluid (CF) exhibits many valuable biological properties, e.g., antibacterial, antifungal, proteolytic, haemolytic, hemagglutinating, and anticancer effects (Hussain et al., 2021; Ečimović et al., 2021; Sinkora et al., 1993; Russell et al., 1983; Haque et al., 2024).

For several years, our research has focused on understanding the mechanism of action of a biologically active polysaccharide-protein complex extracted from the coelomic fluid of the earthworm *Dendrobaena veneta* and called Venetin-1. The preparation was obtained through several processes, such as isolation of CF using electric shock, separation of coelomocytes, filtration, incubation, and lyophilization. A particularly important step was to deprive the fluid of cytotoxicity to normal cells while maintaining high activity against cancer cells of various lines or fungal cells (Fiołka et al., 2019a; Rybicka et al., 2022).

We have already demonstrated some of the effects of Venetin-1: antifungal properties against *Candida albicans* clinical strains (Fiołka et al., 2019b; Fiołka et al., 2020a) without endotoxicity or cytotoxicity towards normal human skin cells, anticancer properties against non-small cell lung cancer A549 cells (Fiołka et al., 2019a; Rybicka et al., 2022; Fiołka and Rzymowska, 2021a), colon cancer cells (Czerwonka et al., 2021; Czerwonka et al., 2020), and the HeLa cell line (Fiołka et al., 2025a), inhibition of the human 20S proteasome (Czerwonka et al., 2020), and anti-aggregation against human platelets (Poniedziałek et al., 2022; Fiołka and Poniedziałek, 2025b). Our initial studies allowed us to confirm the immunostimulating effect of Venetin-1, which resulted in a patent in *in vitro* studies and a publication presenting *in vivo* studies (Mizerska-Kowalska et al., 2021; Czaplewska et al., 2024).

Although we previously described our compound obtained from *D. veneta* CF as a protein-polysaccharide fraction in several publications (Fiołka et al., 2019b), it is in fact a complex named Venetin-1. Dynamic Light Scattering domain-specific analyses performed using Prometheus Panta showed that Venetin-1 was a single nanoparticle. FTIR and XPS spectroscopy and electron microscopy analyses confirmed that we were examining a repeatable particle structure (Fiołka et al., 2019b). Previous chemical analyses showed that proteins of the lysenin family: lysenin-related protein 2 (LRP2) and lysenin were the components of Venetin-1 (Fiołka et al., 2019b; Fiołka et al., 2020b; Fiołka et al., 2021b). In addition, the complex includes carbohydrates, such as inositol, glucose, galactose, and N-acetylglucosamine (Fiołka et al., 2019b). Previously, a fraction from the coelomic fluid was analyzed by bioautography after electrophoretic separation of native proteins in acidic polyacrylamide gels. Two lytic zones of the *Micrococcus luteus* bacteria were observed, indicating lysozyme-like activity (Fiołka et al., 2019b). The ATR-FTIR spectrum of the compound showed a high similarity (82%) to that of hen egg white lysozyme. Since one of the ingredients of our complex can be lysozyme, we decided to analyse the obtained preparation in terms of this enzyme, which may determine the properties of Venetin-1.

## 2 Materials and methods

### 2.1 Earthworm breeding

**FIGURE 1:**
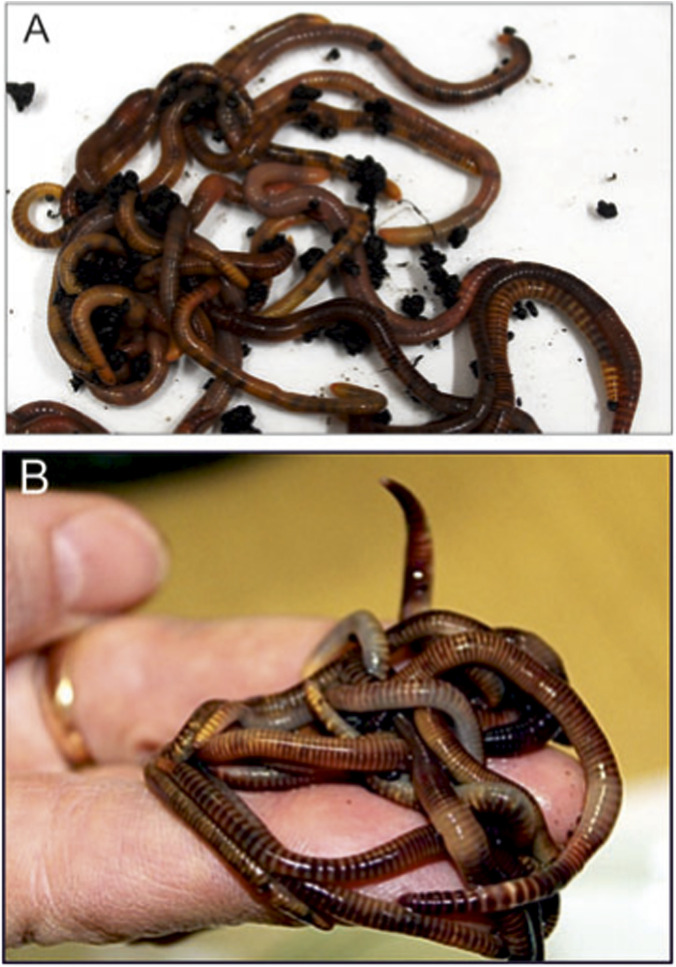
*Dendrobaena veneta* earthworms from a laboratory culture **(A,B)**.

*Dendrobaena veneta* earthworms were kept in a laboratory culture of the Department of Immunobiology of Maria Curie-Skłodowska University in Lublin (Poland) (Figure 1). The invertebrates were kept in plastic containers filled with compost soil at a temperature of approx. 20 °C in the dark. The earthworms were fed with boiled vegetables and green tea leaves twice a week. Pure cellulose, which the animals need to create cocoons, was added to the containers.

### 2.2 Obtaining the Venetin-1 complex

**FIGURE 2:**
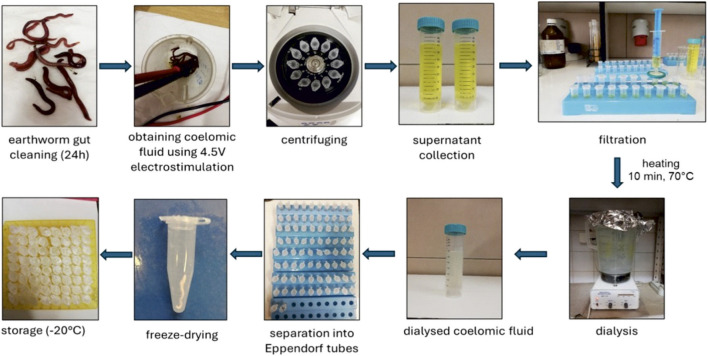
Scheme of obtaining Venetin-1.

The earthworms were removed from the rearing containers and placed in boxes with moist cellulose for 24 h to clean their digestive tract. The animals were then rinsed with sterile water, and excess water was removed by drying on lignin. The CF was collected by mild electrical stimulation (4.5 V) from groups of 10 individuals in 0.9% NaCl (1,500 μL per group) and then centrifuged at 6,000 *g* for 10 min. The supernatant was collected from all Eppendorf tubes into a Falcon tube and then filtered through 0.22 μm Millipore filters to remove possible bacterial and fungal cells. The cell-free filtrate was incubated in a thermal block for 10 min at 70 °C. The obtained fluid was then transferred to a bag with a six to eight or 12–14 kDa cut-off point cellulose membrane. The samples were dialysed in water for 24 h at 4 °C. The fraction obtained after dialysis was transferred to Eppendorf tubes, lyophilized for 24 h, and stored at −20 °C. The scheme for obtaining Venetin-1 is presented in Figure 2. The Bradford method (Bradford, 1976) was used to determine the protein concentration.

### 2.3 Determination of lysozyme activity

The lysozyme type activity of the Venetin-1 obtained using the 6–8 kDa and 12–14 kDa dialysis bags and from the crude CF was determined using the spectrophotometric method against the Gram-positive indicator bacterium *M. luteus*. The effect of lysis of the cell walls of the indicator bacteria was observed as a change in optical density, which was reflected by the discolouration of the bacterial suspension (Shugar, 1952). The test was performed using a lyophilizate of *M. luteus* cells (Sigma) in 33 mM phosphate buffer at pH 6.4 (1 mg mL^-1^). The suspension was boiled to kill the bacterial cells and then cooled to 25 °C with constant stirring on a magnetic stirrer. The preparation with a known protein concentration in the amount of 20 µL was mixed with 700 µL of the bacterial suspension and then incubated in a spectrophotometer. The rate of hydrolysis of the cell walls was measured at 450 nm using a Bio-Rad spectrophotometer after 30 s of incubation at 25 °C. Lysozyme activity was calculated in units of enzymatic activity U/1 mg protein. A unit of activity was defined as a change in absorbance of 0.001 within 1 min at a given temperature. The protein concentration was estimated using the Bradford method (Bradford, 1976).

### 2.4 Determination of lysozyme with the ELISA method

The Human LZMc (Lysozyme C) ELISA kit (Fine Test, China) was used to determine the amount of lysozyme in the fractions and in Venetin-1. The kit uses a Competitive-ELISA detection method (Hemrika et al., 1989). The microplate provided with the kit was pre-coated with anti-LZMc antibody, and the test sample or the standard was completely mixed with a fixed amount of a biotin-labelled LZMc working solution and added to each well of the plate. LZMc contained in the test sample or the standard competed with biotin-labelled LZMc for binding to the pre-coated antibody on the microplate, and free, unbound components were washed away. After adding the HRP-streptavidin conjugate (SABC), the unbound conjugate was washed away. A TMB substrate solution was added, and then TMB was catalysed by HRP to produce a blue product, which turned yellow after adding the stop solution. The OD absorbance at 450 nm was read in a SPECTROstar Nano microplate reader (BMG Labtech). The OD450 value of the sample was then compared to a standard curve using curve fitting software (MARS Data Analysis Software) to determine the concentration of LZMc in the sample. The concentration of lysozyme was inversely proportional to the OD 450 nm value.

### 2.5 Polyacrylamide gel electrophoresis

SDS-polyacrylamide gel electrophoresis (SDS–PAGE) was performed with the method developed by Laemmli (Laemmli, 1970) in 12% acrylamide gels. Samples containing 10 μg and 20 µg of protein were heated at 100 °C for 8 min in sample buffer. Protein bands were detected by staining with Coomassie Brilliant Blue R-250 (Sigma). BlueStar PLUS Prestained Protein Markers (Nippon Genetics Japan) were used.

### 2.6 Immunoblotting

Protein samples at 10 and 20 µg/slot were separated by SDS–PAGE and electroblotted onto PVDF membranes (Millipore) for 90 min at 350 mA. The transfer was conducted in the Mini Trans-Blot Cell Module (Bio-Rad) using a cooling insert. The temperature was equalised by placing the apparatus on a magnetic stirrer. The membranes were blocked with 3% non-fat dry milk in TBS buffer (10 mM Tris-HCl pH 7.4, 0.9% NaCl) for 1 h with gentle orbital shaking at RT and, after washing with TBS, incubated overnight at 4 °C with rabbit primary antibodies diluted in TBS with 3% non-fat dry milk. Polyclonal antibodies to human lysozyme (1:2000, Biorbyt UK) were used to detect lysozyme. The membranes were then washed three times in TBST buffer (10 mM Tris-HCl pH 7.4, 0.9% NaCl, 1% Triton X-100), followed by incubation with secondary antibodies diluted in TBS with 3% of non-fat milk for 1 h at RT. Goat anti-rabbit antibodies conjugated to horseradish peroxidase (HRP) (1:30 000, Biorbyt, UK) were used as secondary antibodies. After three washes in TBS, chemiluminescent signals were developed using Westar Antares ECL Substrate for WB (Cyanagen, Italy) and visualised with the aid of a Bio-Rad ChemiDocTM MP analyser and Image Lab software (Bio-Rad). The experiment was performed in triplicate.

### 2.7 FTIR analysis

Venetin-1 obtained with the use of dialysis bags with different cut-off points and egg white lysozyme (EWL) were characterised by Fourier transform infrared spectroscopy (FT-IR). The ALPHA II spectrometer (Bruker Optics GmbH & Co. KG, Ettlingen, Germany) was used for the studies. The samples in the form of lyophilizates were analyzed using the ATR (Attenuated Total Reflection) technique on a single reflection monolithic diamond crystal (Smith, 2011). The measurements were performed at room temperature in the spectral range of 4,000–400 cm^-1^ at a resolution of 4 cm^-1^ and with a 24-scan rate for the sample and the background.

### 2.8 Raman spectroscopy analysis

In the analysis of the protein secondary structure of Venetin-1 and EWL, a Raman microscopy system (inVia Renishaw) was used. The measurements were performed using a laser emitting at a wavelength of 785 nm, focused on the samples with a 50x/0.75 microscope objective. The spectral data were calibrated using the Raman band at 520.7 cm^-1^ of a silicon internal reference sample.

### 2.9 Bright-field light microscopy

The preparation was placed on a glass slide in an amount of 2 µL water solution, covered with a coverslip, and imaged. Venetin-1 was observed using the differential interference contrast (DIC) technique with a Nikon Eclipse MA200 (Japan) microscope.

### 2.10 Scanning electron microscopy (SEM) analysis

The morphology of Venetin-1 was observed and documented using a Quanta 3D FEG scanning electron microscope. The preparation was used in the study in a lyophilized form. It was not subjected to standard dehydration and gold sputtering, which allowed the visualisation of its morphology in a natural form.

### 2.11 Cryo-EM and grid freezing

Grids used in the cryo-EM data collection were prepared with a Vitrobot mkIV (Thermo Fisher Scientific). A 3-µL drop of Venetin-1 water suspension was added to a freshly glow-discharged Quantifoil 2/1 Cu mesh 200 grid. Excess fluid was blotted from both sides with blot force 3, blotting time 3 s, and no wait time. The sample chamber was cooled down to 4 °C, and the humidity was 95%. A thin film of the sample on the grid was vitrified by plunge freezing in liquid ethane. After freezing, the grids were clipped and transferred to the microscope.

The Cryo-Electron Microscopy (Cryo-EM) data collection was performed in the cryo-EM Facility of the SOLARIS National Synchrotron Radiation Centre, Jagiellonian University, Kraków (Poland). A Titan Krios G3i cryo-electron microscope (Thermo Fisher Scientific) equipped with a Field Emission Gun, a K3 direct electron detector, and a BioQuantum electron energy filter (both Gatan) was used during the measurements. The imaging system was set to 300 kV accelerating voltage. Imaging was done in the low dose EFTEM mode with an energy slit of 20 eV and the total dose of 40e^−^/Å^2^. The movies (40 frames) were acquired with a magnification of ×105.000, which gives a pixel size of 0.84Å. Fringe Free Illumination and Aberration Free Image Shifts were used during the data collection.

Cryo-EM dataset of 7,614 movies was processed using Cryo-EM Single Particle Ab-Initio Reconstruction and Classification (CryoSPARC 4.5.3) software (Punjani et al., 2017). Imported movies were subjected to motion correction, CTF estimation and manual exposure curation. Around 2% of micrographs were discarded because of unsatisfying CTF resolution estimation, astigmatism or frame motion. Next, a small subset of 357 random micrographs was used for preliminary particle picking with a blob picker set to diameter of 45Å. After picking, nearly 285000 particles were extracted with a box size of 128 pixels and subsequently 2D classified into 50 classes. After a couple of rounds of classification, 10 best classes were selected and used as templates for template picking on the whole dataset. Picked particles were inspected, and duplicated particle picks were removed, giving a set of more than 3.5 million coordinates to be extracted with a box of 128 × 128 pixels. After additional rounds of 2D classification, 685390 particles were selected. Best classes, resembling the most clear projections, were used to generate a C1 Ab-initio model with 57553 particles. The whole subset was then used as input for two subsequent NU-refinement jobs - first with C2 symmetry and the second with D2 relaxed symmetry. Resolution of final density was estimated to 4.87Å, but unfortunately, this value is rather far from structural meaning.

### 2.12 2D electrophoresis

Two-dimensional electrophoresis separated proteins based on their isoelectric points (pI) and molecular weights. For the first-dimension isoelectric focusing (IEF), 120 µg of total protein was rehydrated overnight in a rehydration buffer containing 0.4% ampholytes. The sample was applied to an 11 cm immobilised pH gradient (IPG) strip with a nonlinear pH range of 3–10 (3–10NL). The IEF was carried out until a total of 35,000 V h was achieved.

Following isoelectric focusing, the IPG strips were equilibrated in a two-step process. First, they were incubated for 15 min in an equilibration buffer containing 6 M urea, 2% SDS, 50 mM Tris-HCl (pH 8.8), 30% glycerol, and 1% DTT. This was followed by the second incubation round in the same buffer, replacing DTT with 2.5% iodoacetamide for 15 min.

For the second-dimension separation, the equilibrated strips were placed onto a 12% SDS-polyacrylamide gel, and electrophoresis was performed in constant voltage conditions at 100 V. Following separation, the gels were stained with Coomassie Brilliant Blue G-250 to visualise protein spots. A molecular weight standard, the Unstained Protein Molecular Weight Marker (Thermo Scientific, #26610), was included for reference.

Bands from 1D electrophoresis and spots from 2D electrophoresis (Supplementary Figure S1 in the Supplementary Material) were digested according to the standard in-gel digestion procedure (Shevchenko et al., 2006).

### 2.13 SageELF separation

The fractionation of proteins from Venetin-1 and crude coelomic fluid (DVr) was performed using the automated SageELF system (Sage Science, Beverly, MA, USA) with a 3% SDS-Agarose gel cassette (ELP3010) as previously described (Rybicka et al., 2022). The obtained fractions were subjected to the classical digestion procedure, i.e., the Filter-Aided Sample Preparation (FASP) method with trypsin (Trypsin Gold, Promega) (Wiśniewski, 2019). Finally, purification was conducted following the StageTips method. The prepared samples were sent for LC-MS/MS analysis (Rappsilber et al., 2007).

### 2.14 Liquid chromatography and data-dependent (DDA) mass spectrometry of 1D and 2D gel-separated Venetin 1

Mass spectrometric analysis of 2D gel-derived spots was carried out using a TripleTOF 5,600+ system (Sciex, Framingham, MA, USA) coupled with an Eksigent Ekspert MicroLC 200 Plus chromatography platform (Redwood City, CA, USA). Peptide separation was achieved on a ChromXP C18CL analytical column (3 μm particle size, 120 Å pore size, 150 × 0.3 mm dimensions). Peptides were eluted from each sample using a linear gradient ranging from 11% to 42.5% of mobile phase B over 60 min (mobile phase A: water with 0.1% formic acid; mobile phase B: acetonitrile with 0.1% formic acid). The entire system operation was managed using SCIEX Analyst TF software version 1.7.1.

Spectral data acquisition was conducted in the data-dependent acquisition (DDA) mode. Each DDA cycle consisted of a 100 ms survey scan across the 400–1,200 m/z range, followed by MS/MS fragmentation of the top 20 most intense precursor ions, each with an accumulation time of 50 ms and scanned across a 100–1800 m/z range. The resulting cycle time was approximately 1.15 s. Dynamic exclusion was employed to prevent repeated analysis of previously fragmented precursors.

Proteomic profiling of proteins separated by 1D electrophoresis was performed using a ZenoTOF 7,600 mass spectrometer (Sciex, Framingham, MA, USA) coupled with the SCIEX M5 MicroLC-TE system (PAL3 AutoSampler - PAL3 AutoSampler Redwood City, CA, USA). Peptide separation was performed in the trap-elute mode on a ChromXP C18 column (50 mm length, 3 μm particle size, 90 Å pore size, 0.3 mm internal diameter). For each sample, chromatographic separation was achieved using a linear gradient ranging from 5% to 45% of mobile phase B over a 20-min run (mobile phase A: water with 0.1% formic acid; mobile phase B: acetonitrile with 0.1% formic acid).

Instrument control and data acquisition were managed via OS SCIEX 3.4.0.19154 software tailored to the ZenoTOF 7,600 platform. Spectral data were collected in the data-dependent acquisition (DDA) mode, utilising high-resolution MS survey scans to select precursor ions across the m/z range of 400–1,200. The top 50 most intense precursor ions were subjected to MS/MS fragmentation with an acquisition window of 100–1800 m/z, and dynamic exclusion helped to minimise redundant analysis. The Zeno trap technology ensured enhanced sensitivity and duty cycle efficiency, contributing to increased depth in peptide identification.

### 2.15 Liquid chromatography and data-dependent (DDA) mass spectrometry of SageELF fractionated tryptic digests of coelomic fluid and Venetin-1

Evaporated fractions of tryptic digests were dissolved in 30 µL of a loading buffer (0.08% (*v/v*) trifluoroacetic acid (TFA) and 2.5% (*v/v*) LC-MS acetonitrile (ACN) in LC-MS water). Approximately 1 µg of peptides from each fraction were injected and concentrated on an Acclaim™ PepMap™ 100, 5 µm particle size, 1 mm inner diameter, 5 mm length C18 trap column (cat. no: 16045, Thermo Scientific, MA, USA). The trapped sample was desalted by the loading buffer at a constant mobile phase flow of 5 μL/min for 10 min. Analytical peptide separation was performed on PepMap™ 100, 2 µm particle size, 0.075 mm inner diameter, 250 mm length C18 analytical column (cat. no: 164941, Thermo Scientific, MA, USA) by a gradient of mobile phase B (0.1% formic acid (FA) in ACN (*v/v*)) non-linearly increasing its proportion in mobile phase A (0.1% FA in water (*v/v*)). Analytical peptide separation was held at a constant flow of 300 nL/min and started at 2.5% B linearly increasing up to 35% B within 80 min, followed by a 15 min additional linear increase up to 60% B in A. A column flush was initiated by increasing B to 95% in A over 5 min and was kept constant at this setting for another 5 min. Finally, the percentage of B in A dropped to 2.5% during 5 min and was held constant over the next 10 min. A nano-electrospray ion source (Thermo Scientific, MA, USA) was used for ionisation of peptides and their subsequent introduction into an Orbitrap Exploris™ 480 mass spectrometer (Thermo Scientific, MA, USA).

A single replicate of each fraction was measured in the DDA acquisition mode on an Orbitrap Exploris™ 480 mass spectrometer. Each cycle was initiated by a full scan which was operated in a profile mode at 120000 resolutions. A precursor range was set from m/z 300 Th to m/z 1,650 Th. The normalised AGC target was set to 300% with 100 msec maximum injection time. The precursor peptide filter was set to include precursor ions within a charge state ranging from +1 to +6 and the intensity above 3.0e3. The top 20 most intense precursor ions were isolated, and their MS/MS fragmentation spectra were recorded followed by their subsequent exclusion for 20 s with 10-ppm mass tolerance. Precursors were isolated in 1.6 Th wide windows. The normalised collision energy type with the fixed collision energy mode was selected. The HCD collision energy was normalised and set to 30%. MS/MS spectra were recorded at 60000 resolutions. The normalised AGC target was 100%, and automatic setting was used to control the maximum injection time. The centroid data type was selected.

### 2.16 Electrokinetic analysis of Venetin-1 and lysozyme

Electrophoretic mobility, conductivity, and zeta potential were measured for a 10^−3^ mol/dm^3^ KNO_3_ and KCl solutions using a Zetasizer Nano-ZS instrument (Malvern). Since the κa value was around 150, Smoluchowski’s equation was used to calculate the zeta potential. Venetin-1 or lysozyme (EWL) was added to the solution at a concentration of 100 ppm. The mixture was then dispersed using an ultrasonic probe (Sonicator XL 2020; Misonix). After that, the suspension was transferred into 125 mL flasks, and the pH was adjusted to values between 2 and 11 using 0.1 mol/dm^3^ HNO_3_ and KOH solutions. For each sample, five measurements of electrophoretic mobility, conductivity, and zeta potential were taken. The results are shown in the diagrams below. Potassium nitrate (KNO_3_) was used as an electrolyte for zeta potential measurements. It was chosen due to its chemical inertness and lack of specific interactions with the tested compound (Venetin-1). KNO_3_ facilitates precise control of the ionic strength of the solution, which is important for the analysis of electrokinetic properties. Additionally, this electrolyte does not significantly affect the pH of the solution and does not change the protonation state of the functional groups of Venetin-1 or lysozyme, which allows reliable assessment of its surface charge.

### 2.17 Statistical analysis

The Statistica 13.3 program was used to calculate the arithmetic means of the lysozyme concentration and activity. The experimental results were expressed as arithmetic means with ±SD (standard deviation).

## 3 Results

### 3.1 Lysozyme activity determination

Lysozyme-type activity against *M. luteus* was determined in cell-free crude CF, which was not subjected to any thermal treatment, and in Venetin-1 obtained in two ways using dialysis tubes with a cut-off point of 6–8 kDa and 12–14 kDa. The activity was on average 177.06 U/mg (±10,84) in the crude fluid, 436,89 U/mg (±59,58) in the Venetin-1 after using the cut-off point of 12–14 kDa, and 1,224.62 U/mg (±94,37) in the Venetin-1 after using the cut-off point of 6–8 kDa. Noteworthy is the significantly increased activity of Venetin-1 compared to the crude preparation and the two-fold higher activity in the heated preparation obtained using the dialysis bag with the lower cut-off point. The experiments were repeated three times.

### 3.2 Determination of the lysozyme concentration in the Venetin-1 complex

After fractionation in a dialysis bag with a cut-off point of 12–14 kDa, Venetin-1 was additionally subjected to ultrafiltration using Millipore filters cutting off compounds with a molecular weight of 10, 30, 50, and 100 kDa. The obtained results indicate that, with the increase in the molecular weight of a given fraction, the concentration of lysozyme in the sample increased. The highest concentration of this enzyme (121.70 ng mL^-1^) was noted in the fraction containing compounds with a mass above 100 kDa (Table 1). Comparing the same fraction obtained using a dialysis bag cutting off compounds with a mass of 6–8 kDa, the concentration of lysozyme in the sample was higher and amounted to 155.30 ng mL^-1^. When comparing protein fractions containing compounds with molecular mass below 50 kDa from both dialysis bags, a higher protein concentration was observed using the 6–8 kDa cut-off bag than in the case of the 12–14 kDa cut-off point, and these values were 100 and 48 ng mL^-1^ of lysozyme in the sample, respectively (Table 1).

**TABLE 1:** Lysozyme concentration in Venetin-1 using dialysis bags with cut-off points (c.p.) of 6–8 kDa and 12–14 kDa and ultrafiltration.

Coelomic fluid fraction from *D. veneta*	Lysozyme concentration (ng mL^-1^)
V-1 fraction 10–30 kDa (c.p. 12–14 kDa)	17.23 (±1.16)
V-1 fraction 30–50 kDa (c.p. 12–14 kDa)	32.51 (±1.82)
V-1 fraction >100 kDa (c.p. 12–14 kDa)	121.90 (±5.01)
V-1 fraction >100 kDa (c.p. 6–8 kDa)	160.97 (±2.61)
V-1 (c.p. 12–14 kDa)	130.20 (±5.31)
V-1 (c.p. 6–8 kDa)	155,33 (±4.46)
V-1 fraction <50 kDa (c.p. 12–14 kDa)	48.37 (±2.10)
V-1 f fraction <50 kDa (c.p. 6–8 kDa)	100.63 (±3.12)

### 3.3 Immunodetection of lysozyme

**FIGURE 3:**
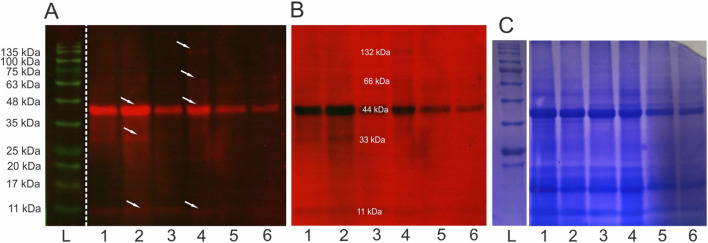
Immunoblotting using anti-human lysozyme antibodies **(A,B)** and electrophoretic separation SDS/PAGE (12%) **(C)** L-ladders, 1-3 Venetin-1 c.p. 12–14 kDa, 4-6 Venetin-1 c.p.6–8 kDa, arrows indicate proteins recognised by antibodies. The obtained images are from the same gel **(C)** and membrane **(A,B)**. Full length blots and gel are available in the Supplementary Material as Supplementary Figure S2.

After the electrophoretic separation and immunoblotting of Venetin-1 with antibodies directed against human lysozyme, proteins with different molecular masses were recognised. The strongest signal came from the band corresponding to a 44 kDa protein, but the antibodies used also recognised proteins with masses of 11 kDa, 33 kDa, 66 kDa, and 132 kDa (Figure 3). For imaging, a multichannel showing proteins as red bands on a black background and vice versa was used to clearly show all recognised proteins. The images from photodetection (Figures 3A,B) were compared with the image of proteins after electrophoretic separation (Figure 3C). Lanes 1, 2, and 3 show separated proteins from Venetin-1 obtained using a dialysis bag with a cut-off point of 12–14 kDa, whereas proteins separated from Venetin-1 obtained with the use of a dialysis bag with a cut-off point of 6–8 kDa are shown in lanes 4, 5, and 6. There are no visible differences in the image between the two samples.

### 3.4 FTIR analysis of Venetin-1

**FIGURE 4:**
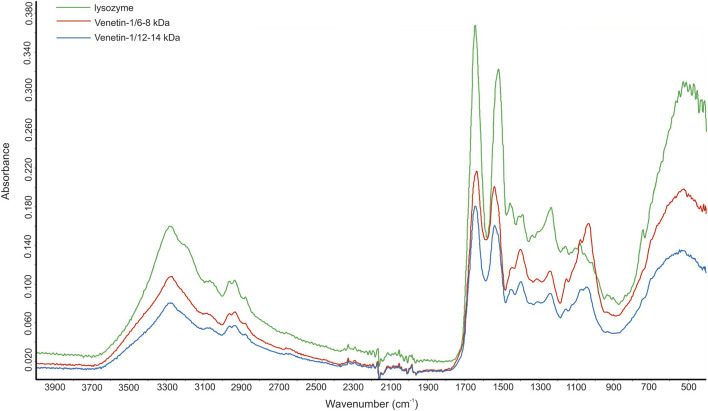
FTIR spectrum of Venetin-1 after dialysis with cut-off point 12–14 kDa (blue line), Venetin-1 c.p. 6–8 kDa (red line), egg white lysozyme (green line).

The FTIR analysis of the Venetin-1 preparation obtained using dialysis bags with a cut-off point of 12–14 kDa (1) and 6–8 kDa (2) and hen egg white lysozyme EWL yielded three spectra, as shown in Figure 4. These spectra differ not only in intensity but also in shape across different spectral ranges. The ATR-FTIR spectrum of compounds 1 and 2 showed high similarity to the spectrum of the egg white lysozyme. The differences in vibrations in the range of 1,040–1,156 cm^-1^ indicated the presence of polysaccharides in the analyzed samples. For all the polysaccharides, the maximum absorbance was found in the range of 950–1,200 cm^-1^, corresponding to C-O-C and C-O-H stretching vibrations characteristic of polysaccharides (Mereghetti et al., 2014). The absorption bands in the range of 1,040–1,070 cm^-1^ indicated the presence of a carbohydrate backbone, while the bands at 1,152–1,156 cm^− 1^ suggest the presence of glycosidic bonds characteristic of saccharide structures (Prabu and Natarajan, 2012). Spectrum 2 exhibits greater similarity to the EWL spectrum in both its intensity and overall profile (Figure 4).

### 3.5 Raman spectroscopy analysis of Venetin-1 and lysozyme

**FIGURE 5:**
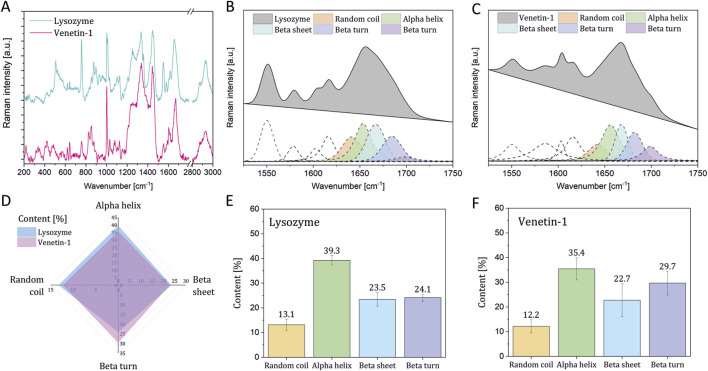
Raman spectroscopy results. Raman spectra of lysozyme and Venetin-1 **(A)** deconvolution of the Amide I band in the Raman spectra of EWL **(B)** and proteins in Venetin-1 **(C)** comparison of the secondary structure content in EWL and proteins in Venetin-1 **(D)** Percentage of secondary structure content in lysozyme **(E)** and proteins in Venetin-1 **(F)**.

The Raman spectra of EWL and Venetin-1 are presented in Figure 5A. The curve-fitting process of the Amide I band was used to determine the percentage content of different types of secondary protein structures in lysozyme (Figure 5B) and Venetin-1 (Figure 5C), such as alpha-helices, beta-sheets, and beta-turns (Ehrlich et al., 2025; Skieresz-Szewczyk et al., 2017; Skieresz-Szewczyk et al., 2019). Figure 5D shows a comparison of the percentage content of specific secondary structures in Venetin-1 and lysozyme, revealing that their area almost completely overlapped. The percentage content of particular secondary structures in Venetin-1 (Figure 5F) was very similar to that of lysozyme (Figure 5E). These results suggest that Venetin-1 may contain lysozyme.

### 3.6 Analysis of Venetin-1 using optical and SEM microscopy

**FIGURE 6:**
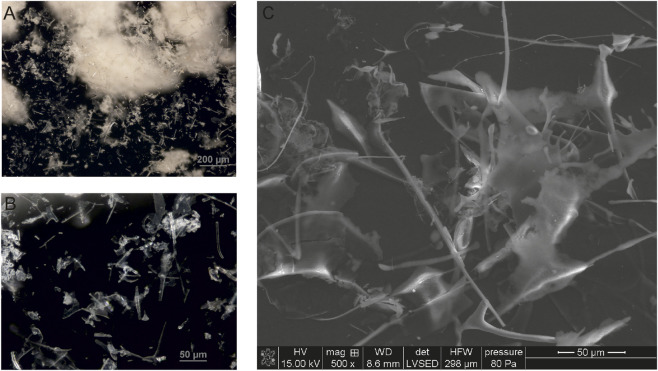
Venetin-1 visualised by optical **(A,B)** and SEM microscopy **(C)**.

The Venetin-1 lyophilizate was observed in an optical microscope using the DIC function. The image shows the observed structures - white aggregates similar to cotton wool balls (Figure 6A). A higher magnification revealed variously shaped structures in the form of needles and resembling ice crystals (Figure 6B). These structures in the same preparation analyzed using SEM can be compared to needles and plates arranged in different planes (Figure 6C).

### 3.7 Cryo-EM analysis of Venetin-1

**FIGURE 7:**
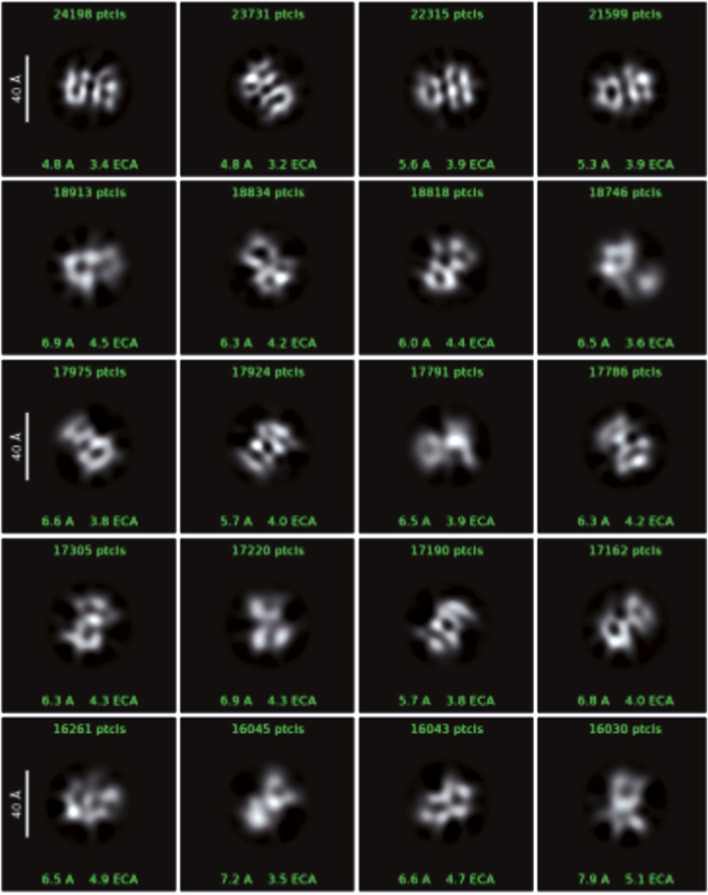
Cryo-EM data showing single particles - 2D classes with box size 128px (scale on 2D classes is 40Å).

**FIGURE 8:**
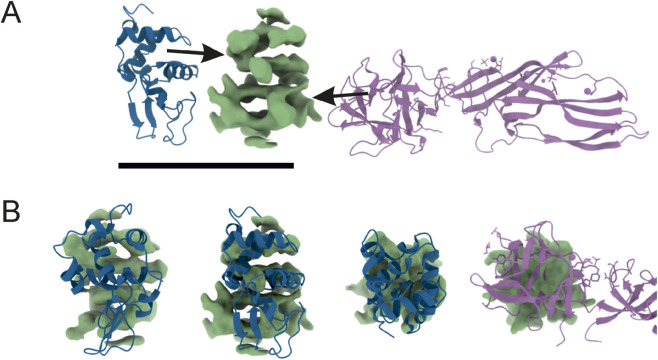
Reconstructed 3D potential density map, resolved at 4.80Å (central, green) next to atomic models taken from PDB **(A)**. On the left: human lysozyme (pdb_00001ip1); on the right: lysenin (pdb_00003zxd). The 3D map was compared in size and shape by overlaying **(B)** with neighbours. The ‘upper’ part of the density resembles part of the lysozyme molecule, and the ‘bottom’ has a size similar to that of lysenin. The final resolution was not sufficient for model building or model docking.

The images obtained during data collection were motion-corrected and averaged to micrographs. After the extraction of millions of particles and interactive 2D classification, the shape of the molecule is emerging as well as secondary structures in the supposed protein fold (Figure 7). The presented 3D model of the Venetin-1 molecule suggests that it contains fragments derived from both lysozyme (blue structure) and lysenin (purple structure) (Figures 8A,B). Regardless of the quick evolution of cryo-EM, the limitations originating from the small size of the molecule are still a relevant problem, prohibiting refinement of 3D potential density maps to nearly atomic resolutions needed for correct atomic model building. The 3D map resulting from the cryo-EM Single Particle Analysis has a resolution of 4.80Å. Unfortunately, this value is rather qualitative, as it is very frequent for small molecules with a weight far below 100 kDa. The final resolution was not sufficient for model building or model docking. The results presented are qualitative and may be subject to a margin of error.

### 3.8 Proteomics analysis

For proteomic analysis, samples of the Venetin-1 preparation were selected and subjected to one-dimensional (three protein bands) and two-dimensional (17 protein spots) electrophoretic separation. Protein identification was conducted using the UniProt database for *Annelida*, supplemented with the lysozyme C sequences from *Homo sapiens* (P61616) and *Gallus gallus* (P00698). These two reference sequences were included based on previous findings reported by Fiołka and collaborators (Fiołka et al., 2019b).

The full identification results are provided in the Supplementary Material. Tables showing in which bands and spots the lysine group proteins and lysozyme were identified together are presented below. Table 2 shows the bands corresponding to the marker masses of 11 kDa - band 1, 33 kDa - band 2, and 44 kDa - band 3, respectively. All forms of lysenins observed in the Venetin −1 preparation are present as well as the main sequence of chicken lysozyme.

**TABLE 2:** Lysenin and lysozyme proteins identified in three bands of the 1D gel. The numerical values in the table correspond to the percentage of sequence coverage for each protein. The full table (Supplementary Table ST2) is available in the Supplementary Material.

Accession	Band 1	Band 2	Band 3	Average mass	Description
O18423	79.12	86.20	48.82	33440.66	Lysenin
A0A2D1PJH7	74.62	80.77	20.00	14460.2705	Lysenin-like protein (fragment)
O18424	16.33	19.67	3.33	33913.3	Lysenin-related protein 1
O18425	83.00	84.00	45.67	34142.535	Lysenin-related protein 2
Q3LX99	25.00	25.00	12.33	33841.24	Lysenin-related protein 3
P00698	22.45	6.12	25.17	16238.643	Lysozyme C
P61626	0.00	0.00	8.11	16537.014	Lysozyme C

In the case of two-dimensional separation, a graphic representation of which with marked spots for analysis is available in the Supplementary Material, lysenins and lysozyme were not identified in encounters 1-4. In the remaining encounters, the presence of lysenin and proteins from this group can be observed: lysenin-like protein (fragment) and lysenin-related protein 2 and 3. In this case, human lysozyme C (P61626) was identified (Table 3).

**TABLE 3:** Partial results showing the identification of lysenins and lysozymes in selected spots from 2D electrophoretic separation. The full table (Supplementary Table ST2) is available in the Supplementary Material.

Accession	Spot 1	Spot 2	Spot 3	Spot 8	Spot 9	Spot 10	Spot 11	Spot 12	Spot 13	Spot 14	Spot 15	Spot 16	Spot 17	Description
O18423	22.56	3.70	6.40	7.07	3.70	40.07	56.23	29.29	38.05	10.44	31.99	36.03	28.96	Lysenin
A0A2D1PJH7	0.00	0.00	0.00	17.69	0.00	40.00	66.15	26.92	34.62	7.69	0.00	0.00	0.00	Lysenin-like protein (fragment)
O18425	32.33	0.00	6.33	9.67	0.00	37.00	56.00	34.67	56.67	10.00	36.00	37.67	26.67	Lysenin-related protein 2
Q3LX99	7.33	0.00	2.67	2.67	0.00	12.67	17.33	2.67	7.33	0.00	11.00	11.00	13.67	Lysenin-related protein 3
P61626	35.14	18.92	8.11	8.11	18.92	18.92	24.32	16.22	28.38	0.00	8.11	29.73	0.00	Lysozyme C

The separation of the coelomic fluid and Venetin-1 in the SageELF system, which allows elution of proteins after separation, facilitated a comparison of the relative presence of lysozyme in different fractions depending on the size. Table 4 shows the results for the coelomic fluid, where both lysozyme sequences (chicken and human) were identified, with the sequence for *Gallus gallus* having a higher percentage of sequence coverage. The separation of Venetin- 1 in the same system yielded weaker results (Table 5). The percentage of sequence coverage was lower, and lysozyme was visible only in a few bands corresponding to different masses.

**TABLE 4:** Lysenin-group proteins and lysozyme identified from isolated earthworm coelomic fluid separated in the Sage ELF system. The full table (Supplementary Table ST3) is available in the Supplementary Material.

Accession	Band 1	Band 2	Band 3	Band 4	Band 5	Band 6	Band 7	Band 8	Band 9	Band 10	Band 11	Band 12	Average mass	Description
O18423	86.20	87.88	86.87	78.79	81.48	88.89	84.51	93.27	85.52	69.02	47.81	37.04	33440.66	Lysenin
A0A2D1PJH7	67.69	60.77	68.46	71.54	68.46	73.85	73.85	90.00	90.00	63.08	30.77	7.69	14460.27	Lysenin-like protein (fragment)
O18424	12.67	18.67	16.00	12.00	10.67	15.67	13.67	20.00	17.33	9.00	2.67	0.00	33913.3	Lysenin-related protein 1
O18425	74.00	77.33	79.33	74.33	77.00	79.33	79.33	86.00	85.33	76.33	54.33	37.00	34142.53	Lysenin-related protein 2
Q3LX99	14.00	12.67	0.00	0.00	0.00	0.00	8.33	19.33	16.00	7.33	7.33	6.00	33841.24	Lysenin-related protein 3
P00698	40.14	40.14	40.14	29.93	40.14	40.14	40.14	40.14	40.14	40.14	40.14	40.14	16238.64	Lysozyme C
P61626	22.30	18.24	22.30	0.00	0.00	0.00	12.84	8.11	8.11	0.00	0.00	0.00	16537.01	Lysozyme C

**TABLE 5:** Lysenin-group proteins and lysozyme identified from isolated earthworm Venetin-1 (B) separated in the Sage ELF system. The full table (Supplementary Table ST4) is available in the Supplementary Material.

Accession	Band 1	Band 2	Band 3	Band 4	Band 5	Band 6	Band 7	Band 8	Band 9	Band 10	Band 11	Description
O18423	66.67	59.93	63.64	59.60	40.07	35.02	46.80	62.96	87.88	73.40	44.78	Lysenin
A0A2D1PJH7	67.69	54.62	63.08	62.31	21.54	7.69	23.08	50.77	90.00	73.85	50.00	Lysenin-like protein (fragment)
O18424	12.67	5.00	9.33	9.67	7.00	2.67	7.00	7.33	28.00	13.00	0.00	Lysenin-related protein 1
O18425	66.67	61.00	64.67	64.33	49.67	40.67	48.33	62.67	87.00	77.33	50.33	Lysenin-related protein 2
Q3LX99	18.67	16.33	18.33	12.33	8.00	6.00	8.00	16.33	30.67	18.67	6.00	Lysenin-related protein 3
T1FG52	0.00	0.00	0.00	1.46	0.00	0.00	0.00	0.00	0.00	0.00	0.00	Lysine--tRNA ligase
P00698	8.16	0.00	0.00	8.16	0.00	0.00	8.16	0.00	0.00	0.00	0.00	Lysozyme C
P61626	12.84	0.00	0.00	0.00	0.00	0.00	0.00	0.00	0.00	0.00	0.00	Lysozyme C

**FIGURE 9:**
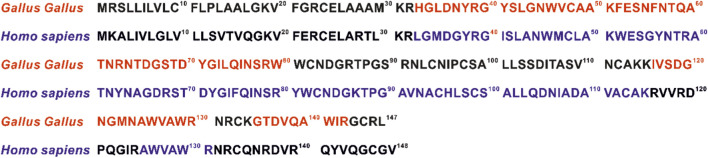
Comparison of human (P61626) and chicken (P00698) lysozyme sequences. Colours (blue *Homo sapiens*, red *Gallus gallus*) indicate parts of the sequence identified in all proteomic analyses.

The collection of information about the sequences identified by the gel analyses, compared with the sequences of both types of lysozyme, is presented in Figure 9. In both cases, the N-terminal fragment corresponding to amino acids 1-32, which also contains the signal fragment 1-16, was not identified. The human lysozyme exhibited the presence of a well-identified fragment 33-115 and one peptide from the C-terminus. For the chicken lysozyme, sequences 32-80 and 116-130 and the C-terminal peptide 135-143 were identified.

### 3.9 Electrokinetic analysis of Venetin-1 and lysozyme

**FIGURE 10:**
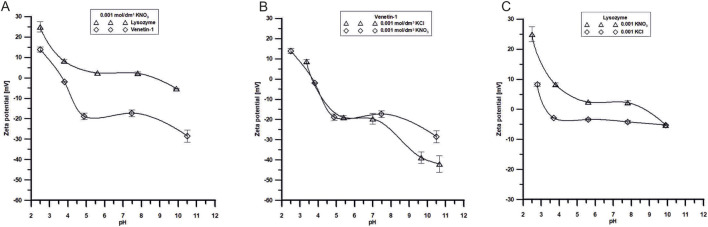
**(A)** Dependence of ζ potential of Venetin-1 or lysozyme in the pH function in 0.001 mol/dm^3^ KNO3 solutions, **(B)** Dependence of ζ potential of Venetin-1 in the pH function in 0.001 mol/dm^3^ KCl and 0.001 mol/dm^3^ KNO_3_ solutions, **(C)** Dependence of ζ potential of lysozyme in the pH function in 0.001 mol/dm^3^ KCl and 0.001 mol/dm^3^ KNO_3_ solutions.

The dependence of the zeta potential on pH for the two studied substances: lysozyme and Venetin-1, in a 0.001 mol/dm^3^ KNO_3_, KCl electrolytes solution is shown in the Figure 10A. The analysis of the data provides insight into the electrokinetic properties of these molecules, which are critical for their colloidal stability. For both systems, a clear decrease in the zeta potential is observed with the increasing pH, indicating a change in the surface charge of the molecules in response to the environmental pH. Lysozyme exhibits positive zeta potential values across a broad pH range, with its isoelectric point (pI), where the zeta potential approaches zero, located around pH ∼10. This suggests that lysozyme carries a net positive charge in acidic and near-neutral conditions, ensuring good colloidal stability at low pH values (particularly at pH 2–4, where |ζ| > 30 mV). In contrast, Venetin-1 displays a lower isoelectric point, around pH ∼5, indicating a different electrokinetic character, probably due to variations in its amino acid composition. At alkaline pH values (above pH 9), Venetin-1 exhibits zeta potential values below −30 mV, suggesting enhanced colloidal stability in basic conditions.

The dependence of the zeta potential on pH for Venetin-1 in the presence of two different supporting electrolytes at identical concentrations (0.001 mol/dm^3^): potassium chloride (KCl) and potassium nitrate (KNO_3_) is shown in the Figure 10B. The data reveal the influence of the electrolyte anion type (Cl^−^ vs. NO_3_^−^) on the electrokinetic behaviour of Venetin-1 particles. For both electrolytes, a general trend of decreasing zeta potential with the increasing pH is observed, indicating a shift in the surface charge of the particles from positive to negative values. This trend is consistent with the pH-dependent ionisation of functional groups on the surface of the biomolecule. The isoelectric point (pI), corresponding to a pH value at which the zeta potential approaches zero, is located in the range of approximately pH 6–7 for both systems. This is a region with minimised electrostatic repulsion and, therefore, the highest risk of particle aggregation. Notably, differences between the two electrolytes become more pronounced at alkaline pH values. In the presence of KCl, Venetin-1 exhibits significantly more negative zeta potential values (reaching approximately – 45 mV at pH 11), suggesting enhanced colloidal stability due to stronger electrostatic repulsion. In contrast, the zeta potential in KNO_3_ at the same pH is less negative (around −35 mV), indicating comparatively weaker repulsive interactions. These differences may be attributed to specific ion effects, particularly the distinct interactions of Cl^−^ and NO_3_^−^ ions with the surface of Venetin-1 particles.

The zeta potential in the lysozyme/KNO_3_ or lysozyme/KCl system refers to the phenomenon of electrophoresis of lysozyme molecules in the presence of two electrolytes. The zeta potential is a measure of the surface charge of particles in a suspension or a solution. Lysozyme is a cationic protein, meaning it has a positive surface charge at neutral pH. Its isoelectric point (pI) is approximately 10; hence, it will carry a positive charge below this pH value and may acquire a negative charge above pH 10. Potassium cations and nitrate or chloride anions interact with lysozyme molecules, affecting their zeta potential, stabilising the suspension, or altering electrostatic interactions between the particles. It can be observed in Figure 10C that, in the lysozyme/KCl system, the zeta potential values are lower throughout the pH range studied, which results from stronger interactions between the electrolyte and the system (Table 6). Such changes can influence the stability of suspensions and protein surface conditioning.

**TABLE 6:** Electrophoretic mobility, conductivity, and pH of Venetin-1 in 0.001 mol/dm^3^ KNO_3_ and 0.001 mol/dm^3^ KCl.

pH	Electrophoretic mobility [ms/Vs]	Conductivity [mS/cm]
0.001 mol/dm^3^ KNO_3_		
2.5	0.75	0.773
3.81	−1.14	0.219
4.88	−1.28	0.212
7.47	−1.41	0.236
10.5	−1.67	0.247
0.001 mol/dm^3^ KCl		
3.36	0.729	0.401
5.39	−1.175	0.252
6.99	−1.262	0.299
9.46	−2.180	0.238
10.66	−2.482	0.357

The addition of the Venetin-1 complex significantly affects the electrokinetic behaviour of the system. Across both KCl and KNO_3_ electrolytes, electrophoretic mobility becomes more negative as pH increases, similar to the baseline behaviour without Venetin-1. However, in the presence of the complex, the absolute values of mobility at higher pH levels are generally lower (less negative) than in the reference solutions, indicating that the surface charge is partially screened or neutralised by the Venetin-1 interaction. This behaviour suggests that the Venetin-1 complex may adsorb onto particle surfaces, modifying their interfacial properties and potentially introducing additional functional groups (e.g., amine, hydroxyl) that influence surface charge. Such adsorption can reduce the zeta potential magnitude and affect the overall stability of the colloidal suspension. Additionally, the differences between KCl and KNO_3_ become subtler in the presence of Venetin-1, probably due to the complex playing a dominant role in determining surface characteristics. Chloride ions (Cl^−^), while still more effective than nitrate (NO_3_^−^) in stabilising surface charge, appear to have a reduced impact in the modified system, possibly due to steric effects introduced by the Venetin-1 layer. Conductivity measurements in the presence of Venetin-1 show slight increases, especially at acidic pH, which may result from the partial ionisation of functional groups on the complex or from improved dispersion leading to greater abundance of free ions in the solution.

The presence of lysozyme in both KNO_3_ and KCl electrolytes leads to a characteristic shift in electrophoretic mobility as pH increases, reflecting the transition of the protein’s surface charge from positive to negative values (Table 7). However, lysozyme exhibits significantly lower absolute mobility values in KNO_3_ than in KCl at equivalent pH levels. This suggests that NO_3_^−^ ions interact less strongly with the protein’s charged surface, resulting in a weaker modification of the electrical double layer. In contrast, Cl^−^ ions from KCl more effectively stabilise the surface charge, which is evident from the markedly more negative mobility values, particularly in alkaline conditions. Conductivity measurements show higher values in KCl solutions across the pH range, consistent with the higher ionic mobility of Cl^−^ compared to NO_3_^−^. Overall, these results demonstrate that the type of background electrolyte plays a key role in determining the electrokinetic properties of protein-containing systems.

**TABLE 7:** Electrophoretic mobility, conductivity, and pH of lysozyme in 0.001 mol/dm^3^ KNO_3_ and 0.001 mol/dm^3^ KCl.

pH	Electrophoretic mobility [ms/Vs]	Conductivity [mS/cm]
0.001 mol/dm^3^ KNO_3_		
2.49	0.6377	0.282
3.79	0.3488	0.238
5.6	−0.1417	0.163
7.8	−0.3744	0.217
10	−0.5257	0.183
0.001 mol/dm^3^ KCl		
2.46	0.749	0.501
3.89	−1.375	0.352
5.81	−1.462	0.399
7.75	−2.280	0.338
10	−2.682	0.457

## 4 Discussion

The cellular mechanisms of invertebrate innate immunity include wound repair, coagulation and clotting responses, phagocytosis, and encapsulation. In addition to these cellular mechanisms, invertebrates possess a wide array of antimicrobial factors, such as lysozyme-type enzymes, proteases, cytolytic proteins, antimicrobial peptides, and enzyme activation cascades. Humoral immunity involves lectin-like molecules that recognise molecular patterns (Cooper and Roch, 2003; Bilej et al., 2010). Earthworm lysozyme belongs to the type *i* of invertebrate lysozyme. This type of lysozyme was first identified in the starfish *Asteria rubens* (Jollès and Jollès, 1975) It is different from all other types of lysozymes (Ito et al., 1999) and occurs in molluscs (bivalves and shells), annelids (earthworms and leeches), echinoderms (starfish), and nematodes (*C. elegans*). The molecular weight of the lysozyme of the earthworm *Eisenia fetida* has been estimated at 13 kDa (Joskova et al., 2009). The invertebrate lysozyme type found in the earthworm *E. andrei* showed high homology with other invertebrate lysozymes–the highest with the enzyme isolated from the medicinal leech (Fiołka et al., 2012a). The antimicrobial mechanism of earthworm CF is associated with, among others, lysozyme-type proteins (Fiołka et al., 2010). In the earthworm, lysozyme occurs in various tissues. In previously published studies, lysozyme in *D. veneta* was also analyzed in coelomocyte extract and cocoon homogenate and, in both cases, native electrophoresis followed by bioautography identified three zones of activity against the indicator bacterium *M. luteus*. The activity and occurrence of lysozyme in the gut of this earthworm were also analyzed and the highest activity was detected in the midgut. Lysozyme in midgut tissues was identified by immunocytochemistry using anti-lysozyme antibodies (Fiołka et al., 2010). A symbiotic bacterium *Raoultella ornithinolytica* with metabolites having lysozyme-type activity: antimycobacterial against saprophytic strains, antifungal against *C. albicans*, and anticancer against breast ductal carcinoma (line T47D) and the endometrioid ovarian cancer line (TOV-112D) was isolated from the intestine wall of the earthworm *D. veneta* (Fiołka et al., 2012b; Fiołka et al., 2013; Fiołka et al., 2015a; Fiołka et al., 2015b). The antimycobacterial activity of lysozyme has been evidenced, and this enzyme is used in laboratory diagnostics (Maier et al., 2024; Asgharzadeh et al., 2015). Its ability to hydrolyse the cell wall of fungi, including yeast, has been described in several articles (Manikandan et al., 2015; Zhou et al., 2017; Hernandez-Tellez et al., 2018). Whether bacterial activity is related to the activity of CF remains an open question.

The analysis of the concentration of lysozyme in fractions of the Venetin-1 nanoparticle showed that dialysis bags with a cut-off of 6–8 kDa retained a larger amount of lysozyme than those with a cut-off of 12–14 kDa, and lysozyme activity was twice as high in the former case than in the latter (Table 1). The FTIR analyses confirmed our observations (Figure 4). Analyses indicate that the monomer mass was approximately 11 kDa. These results correspond to the results obtained using immunodetection technique. The 11 kDa band is visible on the membrane after immunoblotting and was confirmed by MALDI analysis. The remaining bands 33, 44, 66, 132 kDa may correspond to polymers containing fragments lysozyme and lysenin simultaneously. This was also observed in proteomic analyses, where both 2D separation and SageELF gel fractionation showed lysenin and lysozyme proteins. The formation of a larger complex suggests the presence of both proteins at both low and high masses.

Fiołka and collaborators (Fiołka et al., 2012a) demonstrated previously that the antibody directed against human lysozyme recognised proteins of about 14 kDa and 22 kDa found in the crude CF of *D. veneta*. The electrophoretic analysis of native proteins of the CF in acidic polyacrylamide gels with subsequent bioautography revealed two lytic zones of *M. luteus*.

During the preparation of Venetin-1, the cell-free CF was heated to 70 °C. During this time, the preparation lost its lysenin-type pore-forming properties and lost its ability to aggregate red blood cells, becoming non-toxic to normal cells. Lysozyme has the ability to oligomerise during heating. Studies indicate that, in certain conditions, lysozyme can exhibit many other valuable properties. These new properties of lysozyme appear as a result of its modification, which leads to a change in the form of this enzyme from a monomer to a dimer and higher oligomers (Leśnierowski and Cegielska-Radziejewska, 2012). Heat treatment during Venetin-1 preparation is known to promote such protein–protein associations, which may also explain the enhanced lysozyme-like activity we observe compared with crude coelomic fluid. The ∼11 kDa band we detected, corresponds well to the expected monomeric form of earthworm type-i lysozyme. At the same time, the stronger immunoreactive bands at higher molecular masses indicate that lysozyme in Venetin-1 does not remain as a simple monomer. Based on our proteomic results and the cryo-EM density features, we believe these species represent hetero-oligomeric complexes in which lysozyme interacts with lysenin. The abundant ∼44 kDa band is particularly consistent with a 1:1 heterostructure of lysenin (∼33–34 kDa) and lysozyme (∼11 kDa), while the ∼66 and ∼132 kDa bands likely reflect multimeric assemblies of this fundamental unit. The presence of higher-molecular-weight bands does not contradict the ∼11 kDa monomeric mass of lysozyme; instead, it supports the idea that lysozyme in Venetin-1 predominantly functions within a biologically active supramolecular complex.

It has been shown that the modified lysozyme in the new form loses part of its hydrolytic activity and yet gains even more potential. As a result of dimerisation, new activity appears, compensating for the loss of hydrolytic activity and broadening the spectrum of antimicrobial activity of the enzyme (Ibrahim et al., 1996; Ibrahim, 1997; Kijowski et al., 2000; Yang and Leśnierowski, 2019). This new activity is associated with the exposure of the hydrophobic region by exposing tryptophan residues from the interior of the molecule to the outside, which results, among other things, in a greater ability of the enzyme to penetrate the membrane (Ibrahim et al., 1996). Our preliminary analyses of bacteriolytic activity against bacterial species other than *M. luteus* indicate that Venetin-1 was also active against Gram-negative bacteria such as *Escherichia coli*, *Staphylococcus aureus* and *Pseudomonas aerugionosa* and Gram-positive bacteria such as *Bacillus pumilus* and *Bacillus megaterium*.

It has been shown that a drug produced based on the lysozyme dimer has immunostimulating and immunocorrective action (Cegielska-Radziejewska et al., 2008). These assumptions seemed to be confirmed by immunoblotting, where the antibodies recognised proteins with a mass that was a multiple of the oligomers of the recognised lysozyme (Figure 1). In our previous studies on other invertebrates, proteins recognised as lysozymes with a higher molecular mass than the mass of the standard protein were also observed. In the insect *Cameraria ohridella*, an antibody directed against HEWL recognised proteins with a molecular mass of 15 kDa and 28 kDa (Fiołka and Witkowski, 2004). Several forms of lysozyme were observed in snails - in the eggs of the snails *Helix aspersa maxima* and *Achatina achatina* and in the hepatopancreas of *H. aspersa maxima*. In the case of the latter two preparations, the antibodies identified proteins with a molecular mass above 40 kDa (Fiołka and Witkowski, 2004).

A very interesting and noteworthy fact is that anti-human lysozyme antibodies recognise lysozyme in the earthworm preparation, which indicates certain similarity of the lysozyme of this earthworm species to human lysozyme (Table 1; Figure 3). The similarities between Venetin- 1 and lysozyme (EWL) were also demonstrated by the Raman analysis (Figure 5) and the Cryo-EM image comparison (Figure 8). The Cryo-EM studies showed that the molecule could contain both lysozyme and lysenin fragments detected in the MALDI analysis of the nanoparticle (Figures 7, 8). Proteomic analyses confirmed the similarity of *D. veneta* lysozyme to human lysozyme.

Lysozyme is an essential component of the innate immune system in most mammals. Increasing numbers of documented analyses indicate the immunomodulatory effects of lysozymes in combating infections and inflammation. Studies have proved the anti-tumour effects of lysozyme in many types of cancers, possibly through its immunomodulatory effects (Jiang et al., 2021). In humans, lysozyme is produced in neutrophils and macrophages as well as on mucosal surfaces, particularly in type II alveolar epithelial cells in the lung and Paneth cells in the small intestine. Humans have a single lysozyme gene, whereas mice have two closely related genes, lysozyme M and P (Han et al., 2024). Although egg white lysozyme and human lysozyme belong to the same C type, based on crystallographic studies, it has been shown that the basic structural difference between HEWL and human lysozyme is about 30% (Wilken and Nikolov, 2011).

The demonstration of the immunomodulatory properties of lysozyme has attracted considerable attention in the medical community. The antiviral activity of this enzyme can be used concomitantly with immune stimulation in the treatment of gastrointestinal infections (Sava, 1996). The antiviral activity of lysozyme against HIV1 has been described in many scientific articles (Varaldo et al., 1989; Yabe et al., 1993; Lee-Huang et al., 1999; Chen et al., 2018; Kawai et al., 2018). Studies have shown that this enzyme prevents the proliferation of cancer cells, such as human gastric cancer cells *in vivo* (Guo et al., 2007), human lung cancer A549, prostate cancer line LNCap (Jabeen et al., 2017), endothelial cells (ECV304) (Ye et al., 2008), breast cancer cells, and peripheral blood lymphocytes (Jiang et al., 2021; Mahanta et al., 2015). The obtained Venetin-1 nanoparticle shows anti-tumour activity *in vitro* against A549 lung cancer cells (Rybicka et l., 2022), colon cancer cells (Czerwonka et al., 2020), and the HeLa cell line (Fiołka et al., 2025a) (without any cytotoxic effect on normal cells). Venetin-1 also has an immunostimulating effect. It causes the activation of human macrophages *in vitro* and induces the secretion of proinflammatory cytokines, interleukin 6 and interleukin 1β, by these cells (Mizerska-Kowalska et al., 2021). The immunostimulating action was also confirmed in *in vivo* studies on a mouse model (Czaplewska et al., 2024).

Zeta potential is a critical parameter reflecting the colloidal stability and surface charge of nanoparticles. Lysozyme exhibits a positive zeta potential under acidic conditions, ensuring stability at low pH. In contrast, Venetin-1 displays a lower isoelectric point (∼pH 5) and thus shows enhanced stability in alkaline environments. The type of electrolyte anion also modulates electrokinetic behaviour: chloride ions stabilise the electrical double layer more effectively than nitrate ions. Furthermore, incorporation of Venetin-1 into a lysozyme system decreases the overall zeta potential, suggesting adsorption of Venetin-1 onto particle surfaces and consequent modification of interfacial properties. These findings underline the relevance of electrokinetic characterisation for designing stable pharmaceutical formulations containing Venetin-1.

The search for methods to modulate the immune system response has been one of the most important goals of experimental and clinical research in recent years. The development of immunotherapy raises new hopes but also brings disappointments. The methods of immunological treatment used currently often do not bring the expected results in all patients. Therefore, there is still a need to search for new immunostimulants. Research presented on the lysozyme of the Venetin-1 complex is only an introduction to extensive research on demonstrating the immunomodulatory effect of Venetin-1 and its use in medicine. The research results will indicate potential biomedical applications that may help in the development of new chemotherapeutic strategies reducing the risk of immunosuppression. We hope that Venetin-1 can be a factor that increases the body’s natural immunity, supporting treatment when using devastating anti-cancer therapies.

## Data Availability

The data presented in this study were deposited in the PRIDE repository, accession number: PXD063182.

## Supplementary material

SUPPLEMENTARY TABLE S1 Protein identification results performed for 1D electrophoresis and analysed in PeaksStudio software.

SUPPLEMENTARY TABLE S2 Protein identification results performed for 2D electrophoresis and analysed in PeaksStudio software.

SUPPLEMENTARY TABLE S3 Identification results for SageElf fraction of earthworm coelomic fluid analysed in PeaksStudio software.

SUPPLEMENTARY TABLE S4 Identification results for SageElf fraction separations and analysed in PeaksStudio software.

SUPPLEMENTARY FIGURE S1 2D gel electrophoresis with highlighted spots excised from the gel digest and analysed by ESI LC-MS/MS.

SUPPLEMENTARY FIGURE S2
**(A)** full length blot from the Figure 3A. **(B)** full length blot from the Figure 3B. **(C)** full length gel from the Figure 3C.
