# Supplementary figures and images for: Identification of lysozyme in Venetin-1 nanoparticle from the coelomic fluid of the earthworm Dendrobaena veneta
Source: Front Mol Biosci. 2025 Nov 26;12:1719519. doi: 10.3389/fmolb.2025.1719519 (PMC12689324; doi:10.3389/fmolb.2025.1719519)

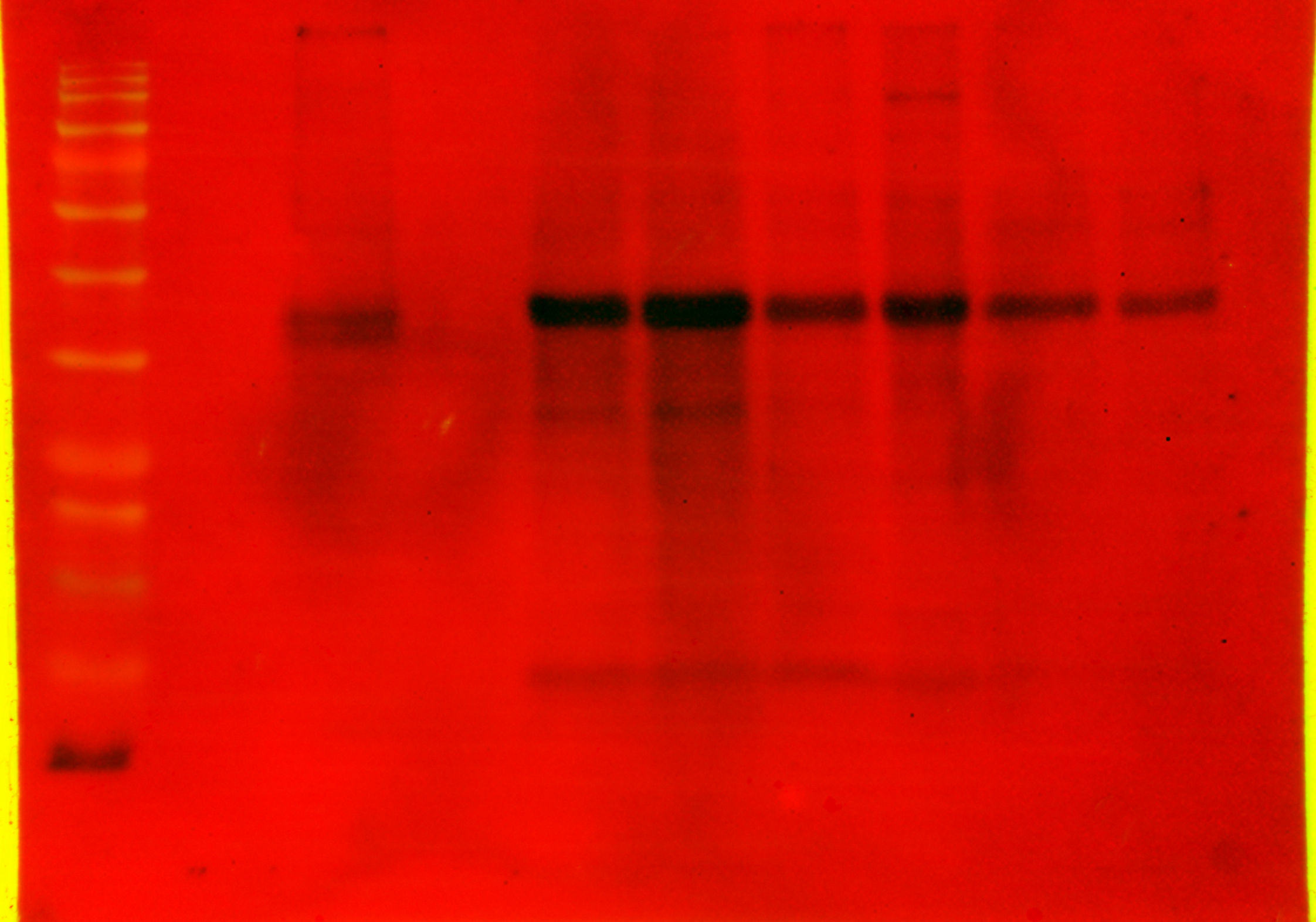

Supplement: Supplementary file 3 [file Image3.tif]

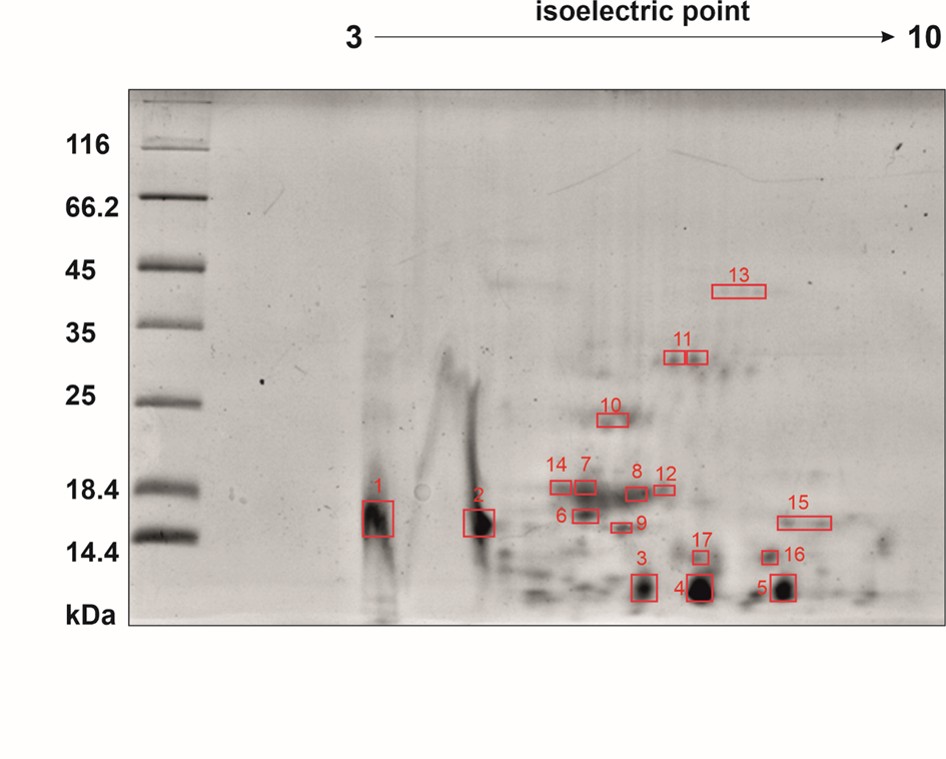

Supplement: Supplementary file 4 [file Image1.jpeg]

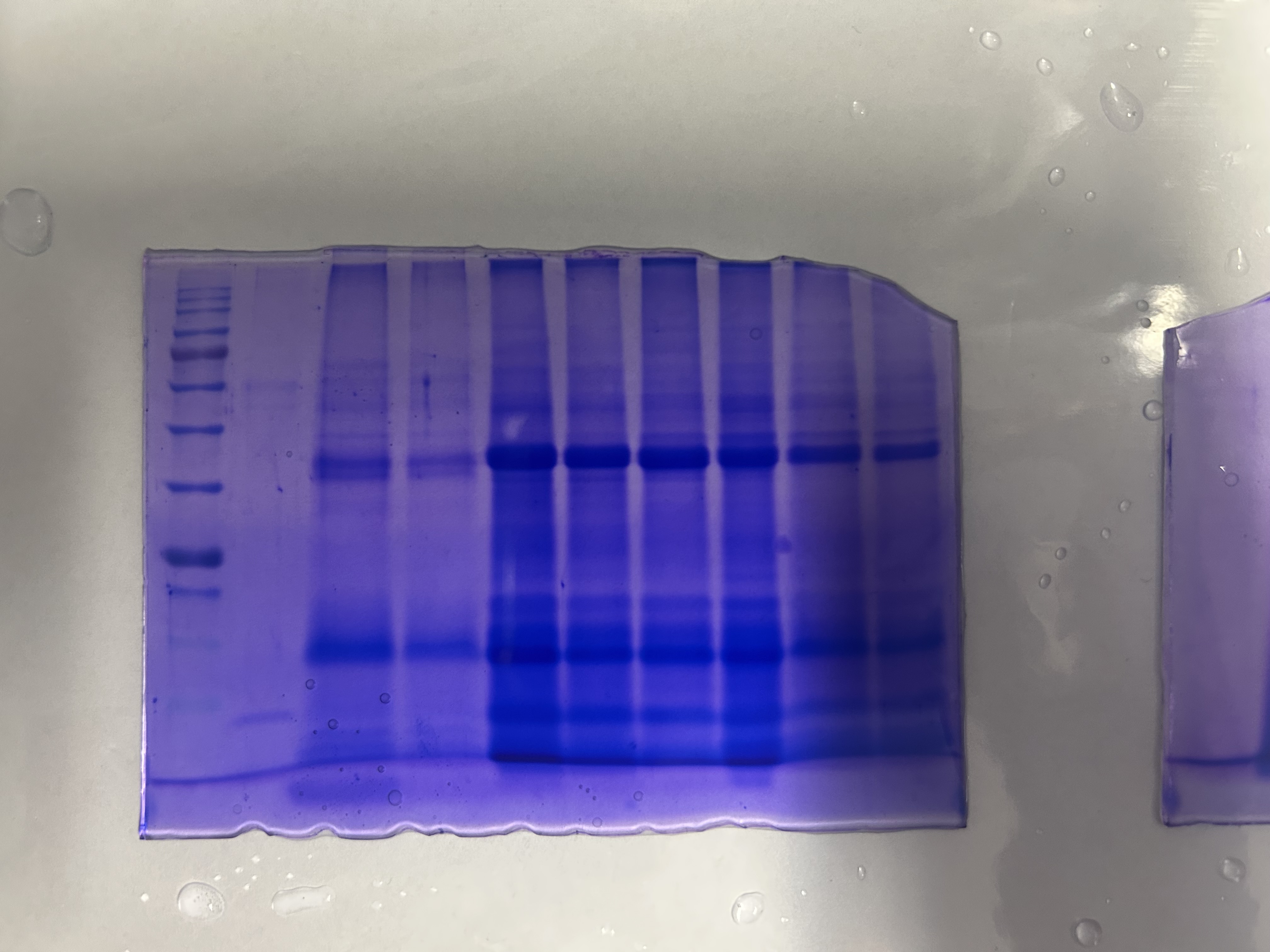

Supplement: Supplementary file 5 [file Image4.jpeg]

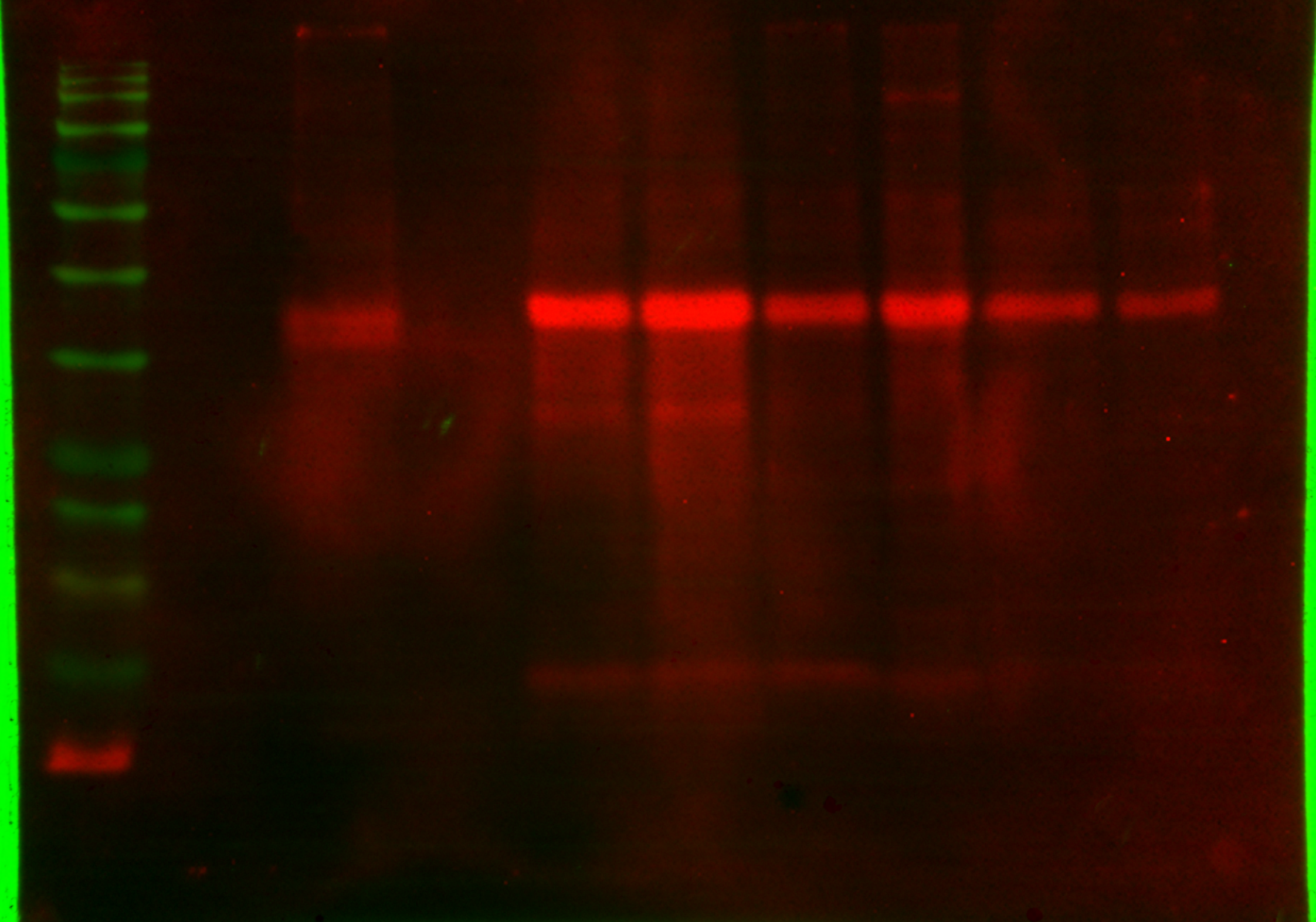

Supplement: Supplementary file 6 [file Image2.tif]
